# Amyloid-β acute exposition affects the CA1 hippocampal network activity and its topological organization, evaluated with multielectrode arrays

**DOI:** 10.3389/frdem.2026.1738954

**Published:** 2026-03-06

**Authors:** David Alcantara-Gonzalez, Elizabeth Santiago, Fernando Peña-Ortega

**Affiliations:** 1 Departamento de Neurobiología del Desarrollo y Neurofisiología, Instituto de Neurobiología, Universidad Nacional Autónoma de México, Querétaro, Mexico; 2 Laboratorio de Biología Computacional, Instituto Nacional de Enfermedades Respiratorias Ismael Cosío Villegas, Mexico City, Mexico

**Keywords:** Alzheimer’s disease, amyloid-β, Estrada index, hippocampal slices, hyperexcitability, neuronal networks, neuronal network topology

## Abstract

**Introduction:**

Neuronal networks enable brain’s information processing through a well-coordinated activity. Disruptions in this activity can impair key brain functions such as synaptic plasticity and long-term memory. Such dysfunctions are relevant to the cognitive deterioration in Alzheimer’s disease (AD). Neuronal circuit alterations in AD are associated with amyloid-β (Aβ) extracellular accumulation across multiple brain regions involved in cognitive regulation. Although several studies have analyzed network topology and examined anatomical, functional, and effective connectivity to understand their role in AD, the direct contribution of Aβ to local neuronal network disturbances has not been investigated.

**Methods:**

We assessed the CA1 hippocampal network structure after acute exposure to Aβ1-42 (30 nM) using an *in vitro* multielectrode array approach. We analyzed neuronal spiking activity recordings, evaluated the frequency of spontaneous synchronized events, and assessed functional connectivity to elucidate the functional alterations in the network. We also elucidated the statistical features of network topology using Graph Theoretical analysis, small-world network properties, and network classification using the Estrada index approach.

**Results:**

CA1 hippocampal neurons showed an average reduced firing frequency. However, some putative pyramidal neurons and interneurons increased their activity. These differences in activity are cell-type-specific, being the interneurons the cells that mainly reduce their firing in presence of Aβ. The number and magnitude of their functional links within the network were not different, but a synchronized firing pattern of different neurons was observed. These changes were associated with alterations to the network’s topological structure, indicating the generation of highly connected nodes in the presence of Aβ.

**Conclusion:**

The main change in the reconfiguration of the CA1 hippocampal network induced by acute exposure to Aβ involved the differential change in firing of different neurons, where the average reduction in firing was found, but some neurons increased their firing. This may constitute an adaptive mechanisms that compensate for neuronal connectivity and help maintain the level of activity. This is the first time the Estrada index has been used to elucidate alterations in the topological neuronal network in an *ex vivo* brain preparation, highlighting its greater sensitivity for detecting changes compared to other topological network analysis approaches.

## 1 Introduction

Neuronal networks are composed of elements, known as neurons, that work collectively to process information. Neuronal networks are mainly described by their anatomical, functional, and effective connectivity (Friston, 2011; Feldt et al., 2011). Network activity generates and maintains brain processes and adaptations, such as synaptic plasticity and long-term memory (Hasselmo and Stern, 2014; Stella and Treves, 2011; Stepan et al., 2012; Jasper, 1936). When this activity becomes aberrant and disrupts the coordinated regulation of widely distributed networks, it can contribute to the progressive cognitive decline observed in Alzheimer’s disease (AD) (Walsh and Selkoe, 2007; Peña-Ortega, 2013; Pena-Ortega, 2019). For instance, alterations in electroencephalographic (EEG) network activity, representing changes in neuronal synchrony, have been observed in patients (Kowalski et al., 2001) and animal models of this illness (Wang et al., 2002). Studies with functional magnetic resonance imaging (MRI) demonstrated hyperactivation of the hippocampal network during memory encoding and impaired deactivation of the default mode network (DMN) in the genetic risk condition (Quiroz et al., 2010; Bookheimer et al., 2000; Anticevic et al., 2012) and in the early stages of AD (Bakker et al., 2012; Dickerson et al., 2005). Furthermore, the hippocampal formation exhibits hypoactivation during memory encoding and no changes in the reduced deactivation of the DMN during later stages of AD (Sperling et al., 2009; Celone et al., 2006). A functional disconnection between the hippocampus and other brain regions (Allen et al., 2007; Flores-Martinez and Pena-Ortega, 2017; Torres-Flores and Pena-Ortega, 2022) has also been reported. Some descriptors of brain connectivity architecture have been used to study AD; these include complexity and entropy measures, graph analysis, and small-world properties, which help assess neuronal networks that have unique features of regional specialization, facilitating efficient information transfer (Vecchio et al., 2016; Sanz-Arigita et al., 2010; Stam et al., 2009; Phillips et al., 2015; John et al., 2016; Sanabria-Diaz et al., 2013; Toussaint et al., 2014; Kim et al., 2015; Lo et al., 2010). Studies have also reported alterations that primarily involve the subnetworks containing temporal lobe areas (John et al., 2016) and suggest that the small-world connectivity pattern can represent a functional complement for hippocampal alterations (Vecchio et al., 2016).

Neuronal circuit alterations in AD are associated with the accumulation of amyloid-β (Aβ)—a neuropathological hallmark of the illness—which generate extracellular aggregates (i.e., senile plaques) in structures involved in the cognitive functional pathway of AD patients, such as the hippocampus and entorhinal cortex (Braak and Braak, 1995; Shankar and Walsh, 2009; Lue et al., 1999). Increased amounts of Aβ, mainly as oligomers, alter information processing at synaptic and neuronal network levels, resulting in cognitive and behavioral changes (Peña-Ortega, 2013; Pena-Ortega, 2019; Naslund et al., 2000). These functional alterations have also been described in cognitively normal individuals with Aβ accumulation (Sperling et al., 2009; Sheline et al., 2010) and in some animal models (Peña-Ortega, 2013; Pena-Ortega, 2019). In the latter, direct Aβ application reduced spontaneous and oscillatory network activity *in vitro* (Adaya-Villanueva et al., 2010; Balleza-Tapia et al., 2010; Gutierrez-Lerma et al., 2013; Pena et al., 2010; Alcantara-Gonzalez et al., 2019) and *in vivo* (Alcantara-Gonzalez et al., 2019; Villette et al., 2010; Colom et al., 2010).

The degree of synchrony among neuronal populations can be determined by measuring and correlating neuronal activity across multiple sites using, for example, imaging or EEG techniques (He et al., 2011; Stam and van Straaten, 2012). However, this approach involves the study of large-scale networks with relatively low spatial resolution, which could lead to a lack of precision in the cellular and spatial properties of the evaluated networks (Chen and Ugurbil, 1999; Burle et al., 2015). Network topology research using higher spatial and temporal resolution approaches, such as multielectrode arrays (MEAs) (Steidl et al., 2006; Negri et al., 2020; Juarez-Vidales et al., 2021; Pinedo-Vargas et al., 2025), could provide more detailed information about smaller, localized cellular networks in the brain. In this regard, MEA studies in AD models have revealed early changes in synaptic function in hippocampal slices (Chong et al., 2011). Additionally, Aβ can reduce the spontaneous activity of hippocampal circuits in culture (Charkhkar et al., 2015; Gortz et al., 2009; Varghese et al., 2010; Schulte et al., 2021; Amin et al., 2017). Nevertheless, no studies have examined the acute effects of Aβ on CA1 local neuronal network activity and topology in brain slices.

In this study, we assessed the structure of the CA1 hippocampal network in the presence of Aβ_1-42_ using the *in vitro* MEA approach. We analyzed neuronal spiking, evaluated spontaneous population events, and assessed functional connectivity to represent the statistical relationship between element activities using correlation values proportional to the connectivity strength (Steidl et al., 2006; Kirkwood, 1979; Nieto-Posadas et al., 2014; Bullmore and Sporns, 2009). Then, we built correlation linkage maps to represent and compare functional configurations of the hippocampal circuit. Finally, we used these correlation values to elucidate the statistical features of the topological network using graph theoretical analysis, small-world properties (Feldt et al., 2011; Bullmore and Sporns, 2009; Stam and Reijneveld, 2007; Boccaletti et al., 2006; Humphries and Gurney, 2008; Telesford et al., 2011), and network classification with the Estrada index approach (Estrada, 2011; Estrada, 2007; Estrada and Rodriguez-Velazquez, 2005; de la Pena et al., 2007). CA1 hippocampal neurons exhibited reduced firing activity over time in the presence of Aβ, which is similar to findings reported in other studies using hippocampal cell cultures (Charkhkar et al., 2015; Gortz et al., 2009). Interestingly, the number and magnitude of the functional links did not change significantly, and a synchronized firing pattern of different elements emerged in the presence of Aβ. These changes were associated with alterations in the network’s topological arrangement, indicating the formation of highly connected nodes in the presence of Aβ. We suggest that the main change in the reconfiguration of the CA1 hippocampal network produced by acute exposure to Aβ involved an adaptive mechanism in which the circuit elements maintained overall activity (homeostatic plasticity) despite the reduction in global circuit functioning. In the long term, this effect could be associated with the generation of specific pathological alterations observed in AD, including early hyperexcitability and susceptibility to seizures (Alcantara-Gonzalez et al., 2019; Alcantara-Gonzalez et al., 2021; Vossel et al., 2017). Furthermore, to our knowledge, this is the first time the Estrada index has been used to elucidate particular alterations in the topological neuronal network in an *in vitro* brain preparation, highlighting its greater sensitivity for detecting changes compared to other topological network analysis approaches.

## 2 Materials and methods

### 2.1 Animals

Experiments were performed using 6–9-week-old male Wistar Rats (*n* = 10) from the animal facility at the Institute of Neurobiology (INB-UNAM). Animals were individually housed at 22 °C, maintained on 12 h light/12 h dark cycles, and given food and water *ad libitum*. All experimental protocols were approved by the local INB-UNAM Animal Experimentation Ethics Committee. Experiments were performed according to the guidelines of the Institutional Animal Care and Use Committee Guidebook (NIH publication 80–23, Bethesda, MD, USA, 1996).

### 2.2 Aβ preparation

Aβ_1-42_ was obtained from American Peptides (Sunnyvale, CA) and underwent an oligomerization process, as previously described (Balleza-Tapia et al., 2010; Klein, 2002). In brief, 1,1,1,3,3,3-hexafluoro-2-propanol (HFIP) was added to solid Aβ_1-42_ to a final peptide concentration of 1 mM, and the mixture was incubated for 60 min at room temperature. HFIP was left to evaporate overnight, and a 5 mM solution was prepared by adding dimethyl sulfoxide. The resultant solution was diluted with F12 medium to reach a final concentration of 100 μM and then incubated for 24 h at 5 °C. Finally, the solution was centrifuged at 14,000 rpm for 10 min at 4 °C, and the supernatant containing the Aβ oligomers, monomers, and some protofibrils was collected and stored at 4 °C until use. We have previously shown that the main Aβ aggregates in this preparation are monomers (4.3–4.5 kDa) and oligomers of low (dimers, trimers and tetramers of ~16–20 kDa) and high molecular weight (globulomers from 37 to 150 kDa), and a very small fraction of protofibrils (150–200 kDa), being the smaller size Aβ aggregates the largest content (Balleza-Tapia et al., 2010). This preparation has been extensively used at a wide concentration range to reproduce Aβ-induced alterations in neuronal activity and behavior (Balleza-Tapia et al., 2010; Alcantara-Gonzalez et al., 2019; Lambert et al., 1998; Alvarado-Martinez et al., 2013; Salgado-Puga and Pena-Ortega, 2015; Salgado-Puga et al., 2015; Salgado-Puga et al., 2017). The final concentration of DMSO in our preparation is too low (0.001%) to cause any relevant biological effect (Tamagnini et al., 2014).

### 2.3 Slice preparation

**Figure 1:**
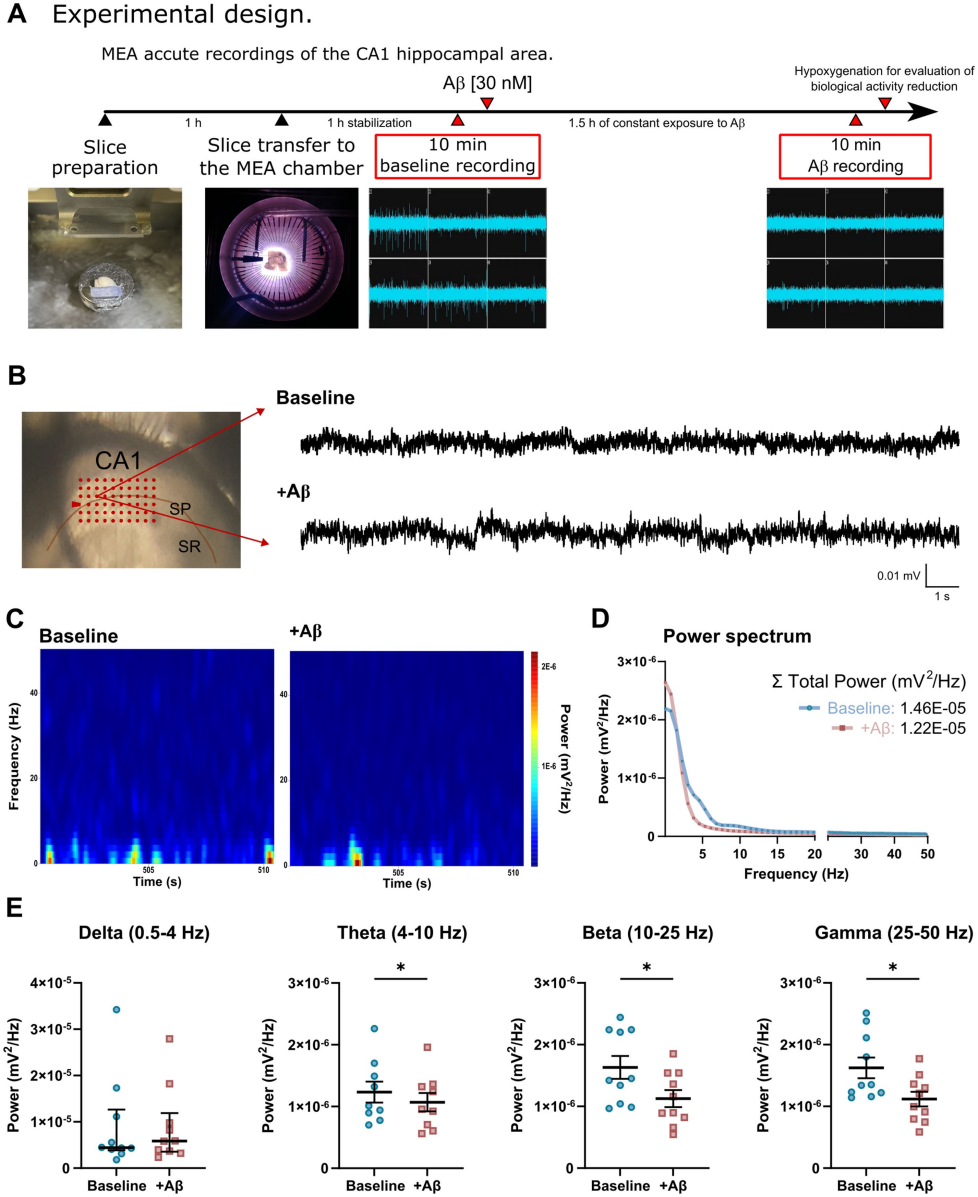
**(A)** The timeline of electrophysiological recordings using multielectrode arrays (MEAs) to determine the effects of acute exposure to Aβ on the spontaneous activity (extracellular action potential generation) of the hippocampal CA1 area. **(B)** Representative image of the CA1 hippocampal slice recordings using a 6x10 MEA matrix (left), and representative raw traces of local field potential (LFP) spontaneous activity recorded under baseline (top) and Aβ (bottom) conditions. **(C)** Ten seconds-long spectrograms during baseline (left) and during acute exposure to Aβ (right) conditions showing the power in color code (mV^2^/Hz). **(D)** Mean power spectrum quantification at baseline (blue) and Aβ (red) conditions. The inset shows the sum of the broadband power. **(E)** Quantification of LFP power in different frequency ranges (Delta: 0.5–4 Hz, Theta: 4–10 Hz, Beta: 10–25 Hz, and Gamma 25–50 Hz) for every individual experiment. Notice a reduction of LFP power in most frequency bands. Values represent the mean ± SEM for parametric data, and median ± interquartile range for non-parametric data. *Paired Student’s *t*-test, *p*<0.05.

Ventral hippocampus (vHC) slices were used for this study because evidence suggests this area has greater intrinsic network hyperexcitability compared to the dorsal portion (dHC) in normal conditions and in AD models (Dougherty et al., 2012; Russo et al., 2021; Papatheodoropoulos, 2015; Spoleti et al., 2022; Masurkar, 2018). Some of these alterations involve changes in neurons intrinsic properties like a more depolarized resting membrane potential and higher input resistance (Dougherty et al., 2012), increased neuron firing rates (Dougherty et al., 2012; Spoleti et al., 2022), dysfunctional sodium and potassium conductance (Spoleti et al., 2022), differences in the dendritic branching pattern (Dougherty et al., 2012), increased sensitivity to epileptogenic stimuli (Papatheodoropoulos, 2015; Derchansky et al., 2004), significantly higher plaque burden (Russo et al., 2021), and a differential distribution of GABAergic receptors (Derchansky et al., 2004; Sotiriou et al., 2005). To obtain ventral hippocampal slices, the animals were anesthetized with an intraperitoneal injection of sodium pentobarbital (50 mg/kg) and transcardially perfused with a cold, protective saline solution containing 238 mM sucrose, 3 mM KCl, 2.5 mM MgCl_2_, 25 mM NaHCO_3_, and 30 mM D-glucose, pH 7.4, and constantly bubbled with carbogen (95% O_2_ and 5% CO_2_). Then, animals were decapitated, and their brains were dissected in ice-cold artificial cerebrospinal fluid (aCSF) containing 119 mM NaCl, 3 mM KCl, 1.5 mM CaCl_2_, 1 mM MgCl_2_, 25 mM NaHCO_3_, and 30 mM D-glucose, pH 7.4, and bubbled with carbogen. One cerebral hemisphere was mounted onto an agar block at a 30° angle, and horizontal 400 μm thick slices were cut from the ventral-middle portion of the hippocampus with a vibratome (Microm HM 650 V, Thermo Scientific, Germany). Slices were allowed to recover in aCSF at room temperature for at least 60 min before any further experimental manipulation (Figure 1A).

### 2.4 MEA recordings

The slices were transferred into a recording chamber at a total volume of 2 mL, which contained the MEA (Multi-Channel Systems; Reutlingen, Germany) at the bottom, ensuring that the CA1 hippocampal layer was always placed just above the MEA (Figures 1A,B, red array, left image). The slices were continuously perfused by recirculating 200 mL of aCSF at a flow rate of ~10 mL/min, constantly bubbled with carbogen to ensure solution oxygenation, and maintained at 30 ± 1 °C with a temperature controller (head-stage heating, Multi-Channel Systems; Reutlingen, Germany). Multiple and simultaneous extracellular recordings were performed using the MEA 2100 system (Multi-Channel Systems, Reutlingen, Germany; Figures 1A,B, raw traces), which comprised a 60-electrode array (TiN/SiN) organized into a 6 × 10 grid with 100 μm interelectrode spacing, 30 μm electrode diameter, and an input impedance between 30 and 50 kΩ. After 1 h of stabilization, a 10-min baseline recording was obtained. Immediately after, the Aβ was bath applied at a final concentration of 30 nM, obtaining a 10 min recording at the end of the 90 min experiment (Figure 1A). The raw data acquired from individual electrodes were digitized at 25 kHz and stored in a personal computer for offline analysis using the MC_Rack software platform (Multi-Channel Systems Reutlingen, Germany).

### 2.5 Power spectrum analysis of local field potential recordings

The raw recordings from three different channels of every MEAs recording (Figure 1B) were analyzed off-line with a classical power spectrum analysis (Gutierrez-Lerma et al., 2013; Alcantara-Gonzalez et al., 2019). The last-minute of each condition was analyzed using a Fast Fourier Transform algorithm with a Hann window in Neuroexplorer (v. 4.126, Nex Technologies, USA). The power spectra (mV^2^/Hz) for the broadband (0–50 Hz) and in the following frequency bands: Delta (0.5–4 Hz), Theta (4–10 Hz), Beta (10–25 Hz), and Gamma (25–50 Hz), were averaged per slice and plotted for each experimental condition. Spectrograms were obtained, using the built-in function in Neuroexplorer (v. 4.126, Nex Technologies, USA). A Wilcoxon signed rank test for non-parametric data, or a paired *t*-test for parametric data were used to compare the power values between Baseline and Aβ conditions.

### 2.6 Spike sorting and frequency analysis

Recordings were analyzed as previously reported (Pinedo-Vargas et al., 2025; Nieto-Posadas et al., 2014). Briefly, raw signals (right raw traces) were pass-band filtered (250–7,500 Hz) using the MC_Rack Software (Multi-Channel Systems, Reutlingen, Germany; filtered traces illustrated in right insert), and then the files of the 10 min baseline and Aβ conditions recordings were merged using the MC_DataTool Software (Multi-Channel Systems, Reutlingen, Germany). Only the filtered channels showing neural activity (spikes) were selected and exported to an offline sorter program (v. 3.3.1; Plexon Inc., USA). Spikes were detected by setting a threshold of 2.6 standard deviation (SD) of the signal. Individual units were distinguished from biological and electrical noise through principle component analysis (PCA) (Super and Roelfsema, 2005) with a semi-automatic approach that used the standard expectation–maximization algorithm from the offline sorter (Juarez-Vidales et al., 2021; Pinedo-Vargas et al., 2025; Nieto-Posadas et al., 2014). When spiking activity could not be classified as originating from individual units (single-unit activity, SUA), we recorded the multiunit activity (MUA) produced by a cluster or group of neurons (Super and Roelfsema, 2005; Bedenbaugh and Gerstein, 1997), thereby raising the detection threshold to 5 SD to be sure this new isolated activity did not include any noise. Spikes that occurred within the refractory period (set as 2 ms) of the selected units or multiunit were discarded. Sorting was verified by a refractory period in the interspike histogram (2 ms) and by auto- and cross-correlation histograms using Neuroexplorer software (v. 4.126, Nex Technologies, USA) (Super and Roelfsema, 2005; Bedenbaugh and Gerstein, 1997). Timestamps for SUA and MUA recordings were used to build raster plots, which were exported to MATLAB (v. R2025a) for further analysis with custom-made routines. We detected the spikes within a 1 ms bin and converted them into binary times of occurrence. To evaluate the synchronous firing between cells (burst), we calculated the sum of all single-cell spike events using 500 ms bins and quantifying all peaks ≥ 5 SD from the noise (root-mean square) of the resulting signal with MATLAB (v. R2025b).

To determine the putative cell identity of the identified SUAs, as putative pyramidal neurons or interneurons, we performed clustering analysis using the spike’s waveform and the firing frequency using a personalized MATLAB routine (v. R2025b). A K-means clustering approach was applied to the data distribution generated by combining the trough-to-peak time (in ms) of spikes belonging to any given SUA with its corresponding firing frequency (in Hz), aiming to differentiate two possible clusters: putative pyramidal neurons (PPyrNs) and putative interneurons (PINs). A normality test and non-parametric comparisons, using a Mann–Whitney U test, were used to compare both groups. Due to the increased number of elements that integrate each group, a Cohen’s D test was applied to determine the size of the effect. To represent the firing frequency of the total number of elements, we averaged the mean values of the different SUA and MUA cell arrangements using Neuroexplorer (v. 4.126, Nex Technologies, USA) and evaluated the proportion of cells that increased, decreased or did not change their firing frequencies. The proportion of putative PyrN and INs that increased and decreased their firing frequency was determined using a personalized MATLAB routine (v. R2025b). We analyzed every element from the two clusters of neurons and evaluated which cell increased or decreased its firing activity in presence of Aβ, in comparison with the baseline period. The frequency change was plotted for the four groups, and the proportion (%) of cells on each group was calculated. A chi-square test was applied to evaluate if the change in activity differs between cell types.

We also assessed the co-occurrence of action potentials between pairs of units using cross-correlation analysis (Kirkwood, 1979; Super and Roelfsema, 2005; Bedenbaugh and Gerstein, 1997; Perkel et al., 1967) with a lag window of ±5 ms, which only includes tight synchrony, discarding any polysynaptic interaction, increasing the likelihood of characterizing networks whit many elements monosynaptically connected. Autocorrelation and cross-correlation functions were normalized to the firing rate to ensure that changes in correlation values were not dependent on changes in firing frequency (Heimer et al., 2002; Nini et al., 1995). Cross-correlation peak values exceeding 5 SD of the correlation noise were considered significant, and those that did not reach this threshold received a value of zero. This cross-correlation threshold selects only “strong connections,” clearly distinguishable from random correlation noise, avoiding synchronized firing likely due to common non-synaptic interactions. Positive and Negative, Binary, and Weighted correlation linkage matrices containing the correlation value of interactions that reached the significance threshold were constructed for every slice in both experimental conditions. For the correlation values, we made the sum of each matrix, and then we compared all sums between groups. We expressed correlation strength as the average of positive and negative weighted correlations in each condition and as the change in the resulting absolute value of subtracting the control correlation matrix from that of Aβ (*Δ* Correlation). Most quantifications included both SUA and MUA, and all data are expressed as mean ± SEM. Statistical differences among groups were tested using a Wilcoxon matched-pairs signed rank test or a paired Student’s t-test (GraphPad Prism 10). Cohen’s D test was used for evaluating effect sizes in the Δ Correlation. For comparing networks according to the Estrada index, an additional Fisher’s exact test was performed considering the number of networks in this classification per experimental condition. Normal distribution of data was analyzed using Shapiro–Wilk test. The power value of *p* < 0.05 was considered as significantly different.

### 2.7 Classification of networks and metrics determination

For topological network analysis, we applied the graph theory approach (Stam and Reijneveld, 2007; Boccaletti et al., 2006; Bullmore and Sporns, 2009), small-world property analysis (Humphries and Gurney, 2008; Telesford et al., 2011), and topological classification based on the Estrada Index (Estrada, 2007; Estrada and Rodriguez-Velazquez, 2005) using MATLAB (v. R2019a) custom-made routines. In general, to reconstruct a neural network applying the graph theory approach, we define a graph from a hippocampal slice, defined as G=(V,E) , where V is a finite group of nodes represented by all the elements identified in each slice by MEAs, and the set of edges ( E) represented by the correlation between them, which happens when the cells activate asynchronously during a certain period of time. According to the graph classification, we considered a connected, undirected, weighted, and unweighted graph with a symmetric relation. We denote n=∣V∣ and m=∣E∣ as the number of nodes and edges, respectively. W is the weighted matrix obtained from correlation measurements, whose values range from zero to one. Adjacency matrices were used to represent the relation between nodes and edges, setting a value of one to any number greater than zero, otherwise it maintains its value of zero.

Once the adjacency matrices were obtained, we computed the statistical metrics as the number of nodes and links (defined above), together with the following:

The *degree* of a node *k_i_* (see Equation 1) is the number of edges incident to the node *i* (Freeman, 1979), and the *average degree* of a graph is the average value of the sum of all degrees in the whole network (Equation 2). Degree distribution is defined as the probability distribution of these degrees across the whole network.


$$k_{i}=\sum_{j}^{n}A_{\mathrm{ji}}$$ (1)

$$K=\frac{1}{n}\sum_{i}^{n}k_{i}$$ (2)

The *density* ρ(G) of an undirected graph G=(V,E) is the ratio of the number of edges found in G with respect to the number of edges in a fully connected graph (Equation 3), where *m* and *n* are the number of edges and nodes in G .


$$\rho (G)=\frac{2m}{n(n-1)}$$ (3)

A *connected component* of an undirected graph is a subgraph where any pair of nodes is connected; that is, there exists a path between each pair of nodes in this subgraph. A graph in which all nodes are connected is considered to have one connected component, namely the whole graph.

The *characteristic path length* is calculated as the average of the shortest paths between all possible pairs of nodes, as expressed in Equation 4. The shortest path between two nodes is $d_{\mathrm{ij}}$ ; its distance is the minimum number of hops from node *i* to node *j*. It is a measure of the efficiency of network information.


$$l(G)=\frac{1}{|V|*(|V|-1)}\sum_{v\in V}\;\sum_{v'\in V,v\neq v'}\;\mathrm{spl}(v,v^{\prime })$$ (4)

The *clustering coefficient* measures how well connected the neighbors of a node *i* are, and is defined by the ratio of existing links between its neighbors and the number of maximum possible links between them (see Equation 5); $m_{i}$ is the number of existing edges between these neighbors and $k_{i}$ is the degree of node *i*. The *average clustering* of the whole network is expressed by Equation 6.


$$CC_{i}=\frac{2*m_{i}}{k_{i}(k_{i}-1)}$$ (5)

$$\bar{\mathrm{CC}}=\frac{1}{n}\;\sum_{i=1}^{n}CC_{i}$$ (6)

To determine *small-world properties,* we compared the clustering coefficient and characteristic path length of our network of interest to those of an equivalent random network (C_rand_ and L_rand_, respectively) for the *σ* index (*γ*/*λ*) (Humphries and Gurney, 2008), as represented in Equation 7. In this equation, C and L are measured to generate the ratios γ = C/C_rand_ and λ = L/L_rand_. These ratios must meet the conditions to classify a network as a small world of C> > C_rand_ and L ≈ L_rand_, resulting in *σ* > 1. We also used another well-known small-world measurement, the *ω* index (Telesford et al., 2011). This measure is determined by comparing the clustering coefficient and characteristic path length of our networks to those of an equivalent lattice network (C_latt_) and an equivalent random network (L_rand_), respectively, as represented in Equation 8. Using the C of an equivalent lattice rather than a random network makes this metric less susceptible to fluctuations. The metric values range from −1 to 1. Values close to zero (from −0.3 to 0.3) are considered small world (L ≈ L_rand_ and C ≈ C_latt_). Positive values indicate more random characteristics (L ≈ L_rand_ and C < <C_latt_), while negative values indicate a more regular graph (L> > L_rand_ and C ≈ C_latt_). Network randomization for calculating σ and ω indexes was performed across ~1,000 networks. The clustering coefficient and characteristic path length were calculated for each network.


$$\sigma =\frac{C/C_{\mathrm{rand}}}{L/L_{\mathrm{rand}}}=\frac{\gamma }{\lambda }$$ (7)

$$\omega =\frac{L_{\mathrm{rand}}}{L}-\frac{C}{C_{\text{latt}}}\;$$ (8)

We also computed the *centrality measures*, defined as follows:

The *betweenness centrality* evaluates a node’s influence with respect to the others by computing the shortest paths between nodes in a network (Freeman, 1979). The most frequent nodes in these paths are significantly more important than others for the transmission of information (see Equation 9). Here, $\sigma_{\mathrm{st}}$ is the total number of shortest paths between nodes *s* and *t*, while $\sigma_{\mathrm{st}}(i)$ is the number of shortest paths that pass through node *i*, for all *s* and *t* nodes that belong to V.


$$C_{B}(i)=\sum_{s\neq i\neq t}\frac{\sigma_{\mathrm{st}}(i)}{\sigma_{\mathrm{st}}},\;\forall s,t\in V$$ (9)

*Closeness centrality* is a global centrality measure that quantifies the ability to reach all nodes from a certain node (Sabidussi, 1966). It is defined as the reciprocal of the total distance from a node to all other nodes (see Equation 10). To compute this metric, we considered an unweighted, undirected graph in which the distance is calculated as the number of hops to reach a node from another.


$$C_{C}(v)=\frac{1}{\sum_{t\in V}d_{G}(v,t)}$$ (10)

*Eigenvector centrality* is a measure to find the most central nodes (Bonacich, 1987). A node is considered important if it has significant neighbors, as measured by means of its score. The score of a node *i* is defined as a proportion of the sum of all its neighboring scores (see Equation 11). In this equation, N(*i*) is the set of neighbors of *i* and λ is a constant (the largest eigenvalue of the adjacency matrix A).


$$x_{i}=\frac{1}{\lambda }\sum_{j\in N(i)}^{n}\;x_{j}=\frac{1}{\lambda }\sum_{j\in V}^{n}\;a_{\mathrm{ij}}\;x_{j}\;$$ (11)

For node *i*, the *eccentricity* measure is the number of hops to the farthest node (Sabidussi, 1966) in the network. This metric also uses the geodesic path (or shortest path) between nodes, where the maximum eccentricity within the network is the diameter and the minimum is the radius.

### 2.8 Topological classes

Finally, to characterize neural network topology, we used an approach proposed by Estrada (2011) and Estrada and Rodriguez-Velazquez (2005) based on the correlation between local and global communication in the network. This approach generates four types of networks, known as universal network classes. The eigenvalues of the adjacency matrix A(G) are the eigenvalues of G and form the spectrum of G. Suppose λ_1_ … λ_n_ is the spectrum of G such that λ_1_λ_2_ ≤ … λ_n_. The Estrada index of graph G is defined as shown in Equation 12.


$$\mathrm{EE}=\mathrm{EE}(G)=\sum_{i=1}^{n}e^{\lambda_{i}}$$ (12)

The Estrada index was used to find odd closed path subgraphs (local communications), and eigenvector centrality was applied to measure global communicability. The correlation between these parameters (i.e., Estrada index and eigenvector centrality) is exemplified in (on a logarithmic scale), where the red dotted line in the middle represents the network’s behavior when both communicability measures behave similarly at local and global levels. According to this approach, nodes on the red line are classified as class I, which categorizes graphs as homogeneous networks (ideal networks). Here, the node degree is equal to the rest of the nodes in the whole network. Upper and lower limits (different for each network sample) are obtained from the correlation values of the ideal line ± a determined threshold, allowing classification of other class types, defined as heterogeneous networks, whose graphs exhibit distinctive variation in the degree distribution of nodes (Estrada, 2007). Thus, class II networks have communicability values below the lower bound. Additionally, this class is characterized by the formation of clusters separated by structural holes —that is, communities with high local communication but low global communication— in the network assembly. Networks are categorized as class III if the correlation exceeds the upper limit. Class III networks are formed by highly connected nodes (central cores) with groups of nodes at the periphery (i.e., communities with low local communication but high global communication). Finally, class IV networks exhibit a combination of the characteristics of classes II and III. We applied this methodology to binary and weighted matrices and implemented certain modifications to improve the characterization of neural network structures. Specifically, we set a new threshold and used weighted correlation matrices.

The networks for the Aβ and baseline control conditions were represented in graphs using Gephi software (Gephi Consortium). The ForceAtlas layout algorithm was used to construct small-world and scale-free networks. All parametric data are expressed as the mean ± SEM and non-parametric as the median ± interquartile range, and statistical differences among groups were tested using paired Student’s t-tests or Wilcoxon matched-pairs signed rank tests. Kolmogorov–Smirnov test was used to compare cumulative distributions. All statistical analysis were performed using GraphPad Prism 10 and Matlab routines. Statistical differences were set at *p* < 0.05. For the comparison of class IV networks according to the Estrada index, an additional Fisher’s exact test was performed considering the number of networks in this classification per experimental condition.

## 3 Results

### 3.1 Acute Aβ exposure decreases the LFP power *in vitro*

To evaluate the changes in the spontaneous CA1 hippocampal activity induced by acute Aβ exposure, we evaluated the LFP power of hippocampal slice recordings using the FFT (Figures 1B–D). We found that the presence of Aβ (*n* = 10) for 90 min did not induce a significant change in the broadband power (0–50 Hz; *p* > 0.05; Figure 1D). However, when the spectrum was separated into the different frequency ranges, we observed a reduced power in the Theta (1.07e-6 ± 1.50e-7 vs. 1.23e-6 ± 1.70e-7 mV^2^/Hz, respectively, *p* = 0.019; Figures 1C,E) Beta (1.13e-6 ± 1.37e-7 vs. 1.63e-6 ± 1.85e-7 mV^2^/Hz, respectively, *p* = 0.007; Figures 1C,E) and Gamma range (1.12e-6 ± 1.18e-7 vs. 1.62e-6 ± 1.68e-7 mV^2^/Hz, respectively, *p* = 0.001; Figures 1C,E), when compared with baseline. The power in the Delta range did not show significant change (*p* > 0.05; Figures 1C,E), although it seems to have a trend to increase.

### 3.2 Acute Aβ exposure affects the hippocampal unit activity

**Figure 2:**
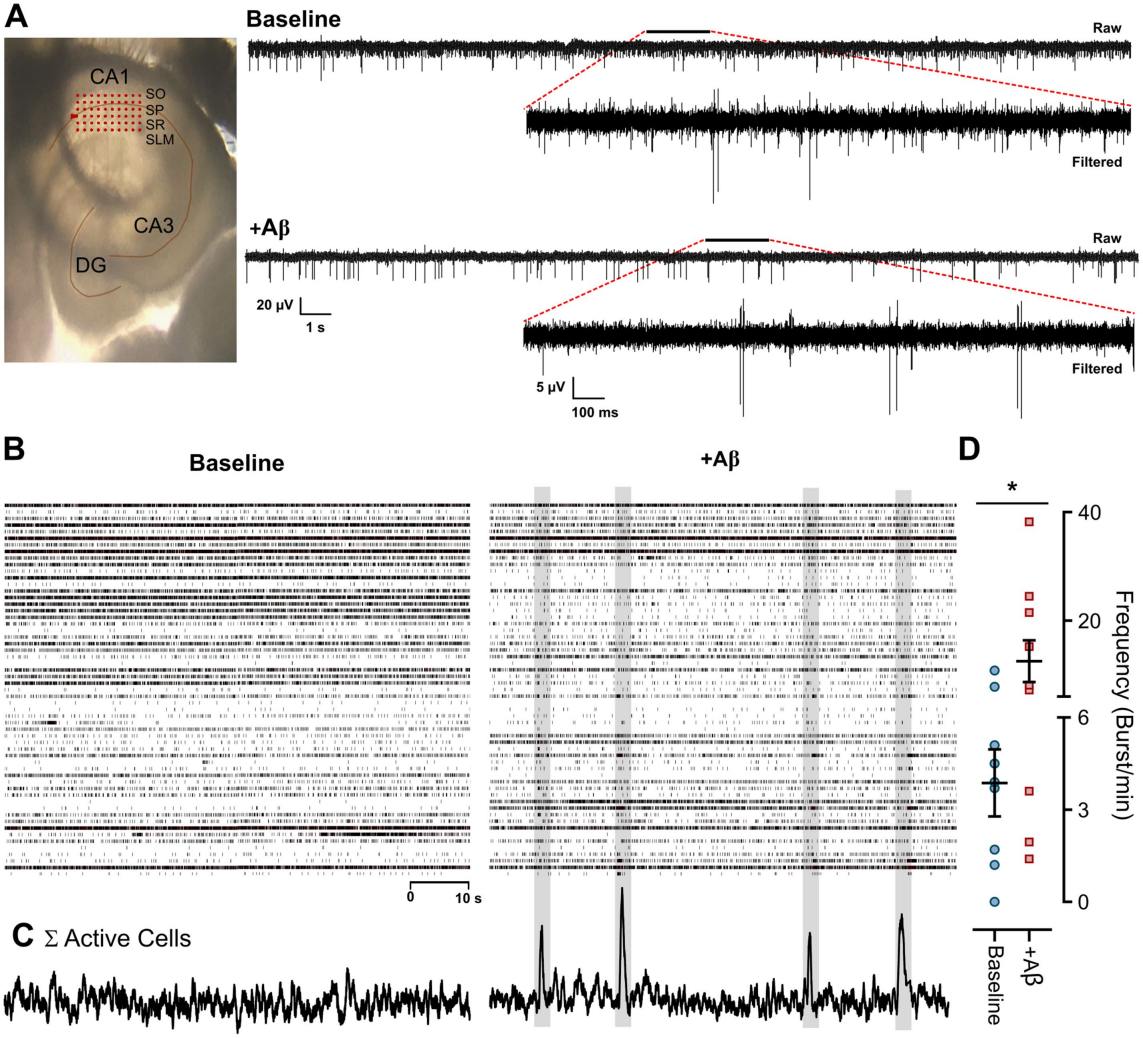
Spontaneous activity recording of the CA1 hippocampal area during acute exposure to Aβ using multielectrode arrays (MEAs). **(A)** Representative image of the CA1 hippocampal slice recordings using a 6×10 MEA matrix (left), and representative raw traces of spontaneous activity, along with high-pass filtered data (insets showing the timeframe indicated by the bar) recorded under baseline (top right) and Aβ (bottom right) conditions. **(B)** Representative raster plots displaying the activity of the hippocampal neuronal elements recorded under baseline (left) and Aβ (right) conditions. **(C)** The sum of all the elements in 500 ms bins and **(D)** the average value of bursting firing activity are shown. Note that the main effect of Aβ is a general decrease in cellular activity and the generation of hypersynchronous events. Values represent the mean ± SEM. *Paired Student’s *t*-test, *p* < 0.05. CA1–3: *Cornus ammonis* regions 1–3; DG: Dentate gyrus; SO, *Stratum oriens*; SP, *Stratum pyramidale*; SR, *Stratum radiatum*; SLM, *Stratum lacunosum-moleculare*.

Recordings from slices placed over a 6×10 MEA (Figure 2A, left) under baseline (Figure 2A; raw, top-right) and Aβ (Figure 2A; raw, bottom-right) conditions displayed the tissue local field potential (LFP) and multiunit activity. These comprise extracellular action potentials of several neurons, as has also been described during MEA evaluations in hippocampal slices (Gong et al., 2010) and organotypic or cellular cultures (Gong et al., 2016; Srinivas et al., 2007). The corresponding high-pass-filtered data of the raw signal (Figure 2A inset, filtered top-right) were generated for each condition, making spikes more evident. The recordings of 10 hippocampal slices resulted in a total of 613 hippocampal units, of which 583 were single-unit recordings (32–88 per slice) and 30 were multi-unitary recordings (2–10 per slice), representing 4.9% of the total units.

In this study, we demonstrated that the presence of Aβ led to a general decrease in some LFP frequency ranges, which was associated with changes in unit firing activity. This effect can be observed in the representative raster plots of hippocampal activity under the Aβ condition (Figure 2B, right graph) and baseline condition (Figure 2B, left graph). Each row displays the activity of hippocampal cells categorized as SUA and MUA, recorded by several electrodes of the MEA. An interesting effect was the presence of hypersynchronicity periods during Aβ exposure. During these periods, several elements fired together (Figure 2C, right graph), as displayed by the sum of active cells in a 500 ms time bin, which was significantly different from the baseline period (12.53 ± 3.85 vs. 3.87 ± 1.09 for Aβ and baseline, respectively; *p* = 0.025; Figure 2D).

### 3.3 Firing frequency of two different putative cell types is affected by acute Aβ exposure

**Figure 3:**
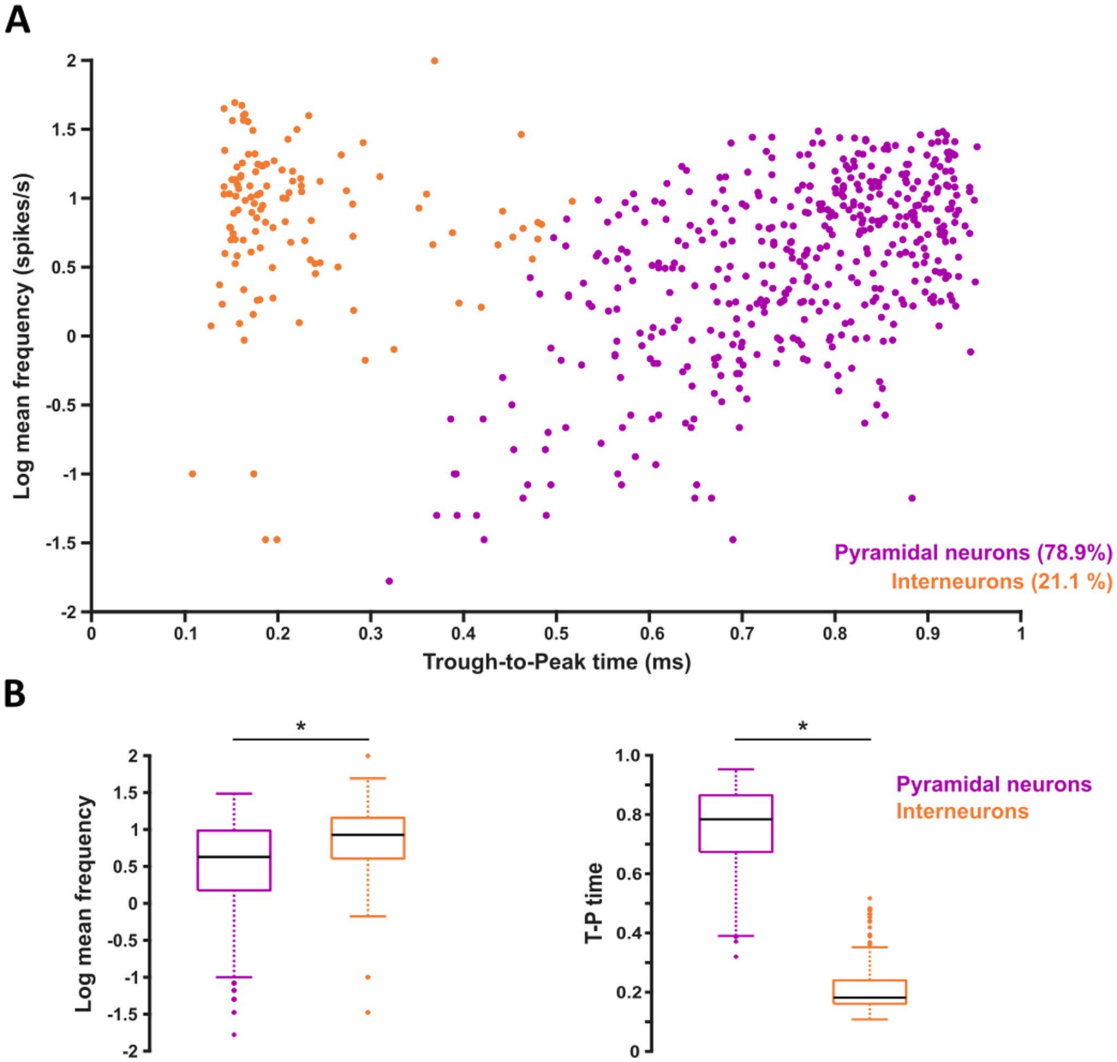
Identification of putative pyramidal neuron or interneuron units. **(A)** Scatter plot for all single units (SUAs) showing the clustering of putative excitatory and inhibitory units based on their mean firing frequency (Hz) and waveform width (through-to-peak duration, ms). A *K*-means clustering analysis was applied to group SUAs into two possible clusters: putative pyramidal neurons and interneurons. **(B)** Quantification of the two variables (log frequency and trough-to-peak time) used in **(A)**. Values represent the median ± interquartile range in box plots. *Mann–Whitney *U* test, *p* < 0.05. A Cohen’s D test was applied to determine the size of the effect.

A K-means clustering analysis of the SUAs data distribution using the trough-to-peak time of their spikes and their corresponding firing frequency (Figure 3) revealed two distinctive populations. One population that includes 78.9% of the total neuronal elements corresponds to putative pyramidal neurons (Lin et al., 2021; Petersen and Buzsaki, 2020; Fernandez-Ruiz et al., 2021). Meanwhile, the other population, that includes 21.1% of the total neuronal elements corresponds to putative interneurons (Lin et al., 2021; Petersen and Buzsaki, 2020; Fernandez-Ruiz et al., 2021) (Figure 3A). When comparing the log frequency value between the two clusters, we found that it is significantly higher in PINs (0.84 ± 0.05) than in PPyrN (0.51 ± 0.03; *p* < 0.001; Figure 3B, left graph). When comparing the trough-to-peak time between the two clusters, we found that it is significantly lower in PINs (0.22 ± 0.01 ms) than in PPyrN (0.76 ± 0.01 ms; *p* < 0.001; Figure 3B, right graph). The Cohen D test for both parameters showed that the size of effect for the trough-to-peak time is large (*d* = 4.24), meanwhile for the firing frequency the size of effect is medium (*d* = 0.52). In both cases, these parameters allowed an accurate separation of elements into two clusters.

**Figure 4:**
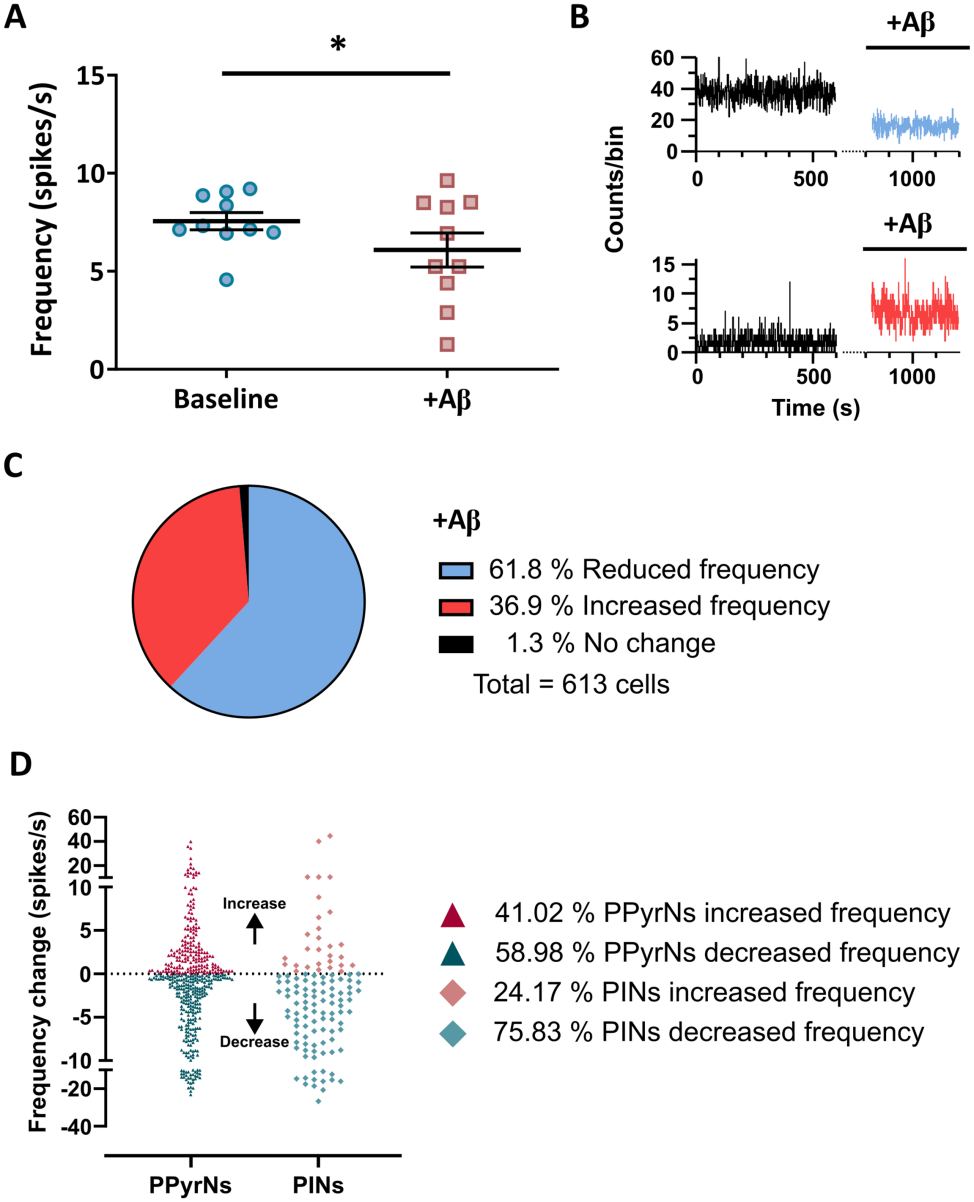
The reduction in global activity induced by Aβ involved opposing changes in the firing frequency of two subsets of neurons. **(A)** Average values of the firing frequency reduction (spikes/s) observed during acute exposure to Aβ. **(B)** Graphic representations of the activity of two types of neurons that reduce (counts/bin, upper traces) and increase (bottom traces) their firing frequency in the presence of Aβ. **(C)** The proportion of cells that changed their firing frequency (total number and % of cells). **(D)** Firing frequency change and the proportion (%) of putative PyrN and INs that increased and decreased their firing frequency in presence of Aβ. Note that Aβ effects involve not only a decrease in cellular activity but also an increase in spontaneous activity in a lesser proportion of cells, which probably reflects adaptive mechanisms. Values represent the mean ± SEM. *Paired Student’s *t*-test; *p* < 0.05.

The presence of Aβ induces an averaged reduction in neuronal firing frequency (from 7.56 ± 0.44 to 6.09 ± 0.87 spikes/s for baseline and Aβ conditions, respectively; *p* = 0.039; Figure 4A). However, two different subsets of neurons were identified in the presence of Aβ. Firing frequency decreased in one subset (Figure 4B, top graph, blue trace) and increased in the other (Figure 4B, bottom graph, red trace). When we estimated the number of cells that changed their activity, we found that of the 613 elements, 61.8% (397 cells) reduced their firing frequency, 36.9% (226 cells) increased it, and 1.3% (8 cells) did not change it (Figure 4C).

The identified PPyrNs and PINs differentially changed their firing frequency in the presence of Aβ (Figure 4D). Namely, 41.02% of PPyrNs and 24.17% of PINs increased their frequency, meanwhile 58.98% of PPyrNs and 75.83% PINs decreased their frequency (Figure 4D). A chi-square test demonstrated a significant association between cell type and direction of activity change (χ^2^ = 6.587, df = 1, *p* = 0.010). PPyrNs showed a higher proportion of activity increases (41.02%) compared to PINs (24.17%), whereas PINs were more frequently characterized by decreased activity (75.83%). These findings indicate cell-type–specific differences in activity modulation. These data highlight the fact that most of the elements from both subtypes reduced their activity in the presence of Aβ, but it is evident that PINs are the most susceptible cell type.

### 3.4 Aβ did not affect the hippocampal unit correlations

**Figure 5:**
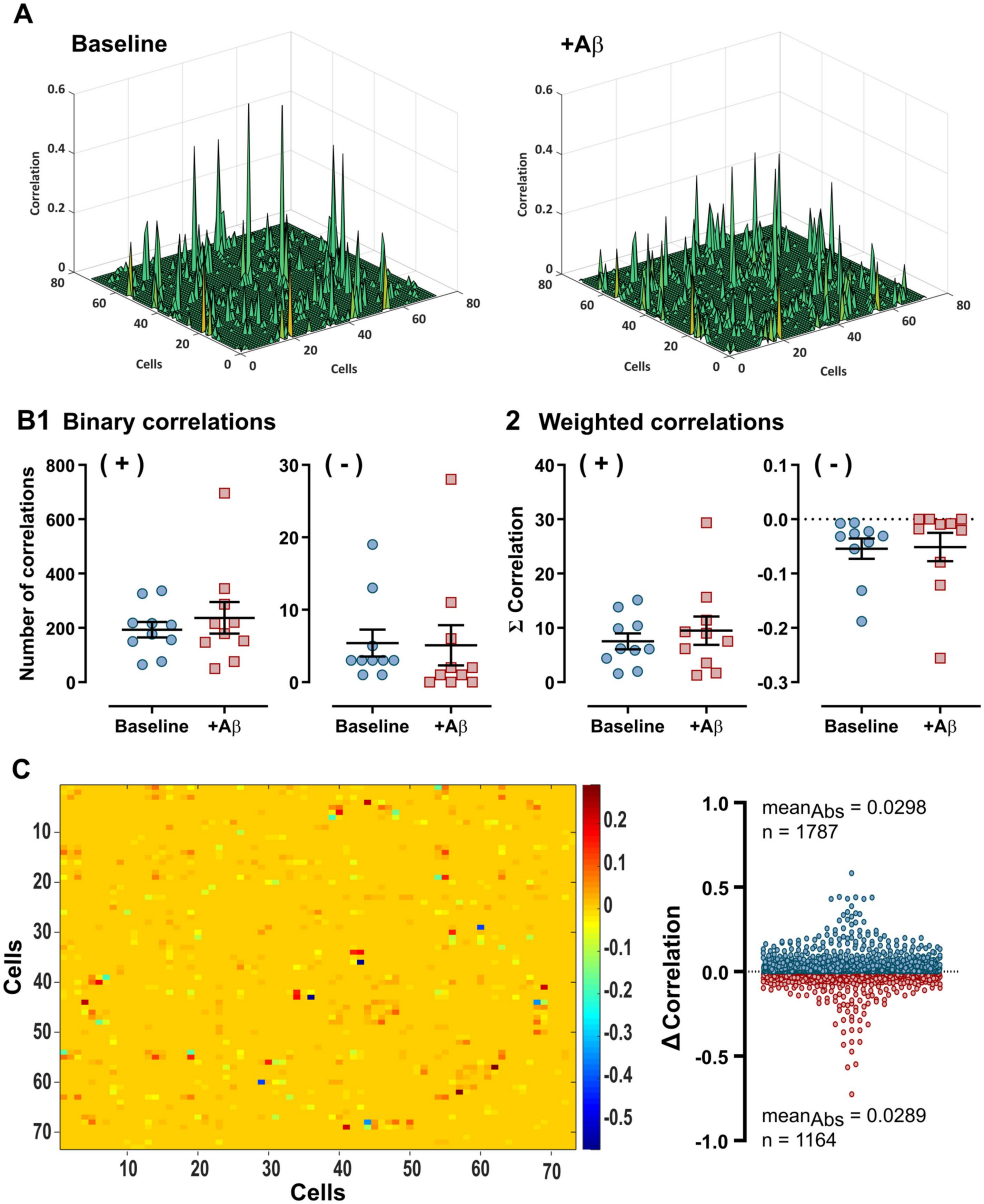
Cross-correlation analysis for the characterization of changes in the positive and negative functional connections within the CA1 hippocampal network. **(A)** Representative cross-correlograms in peak-representation maps of positive correlations indicating the amount (number of peaks, binary) and weight values (height of peaks, weighted) from pairwise analysis between elements with correlation peaks >5 SDs of the noise correlation under baseline (left graph) and Aβ (right graph) conditions. **(B)** The average values of all positive (+) and negative (−) binary (1) and weighted (2) correlations are plotted. **(C)** The difference in correlation (*Δ* Correlation) during Aβ treatment indicates several positive and negative changes in correlation values. Note that the average correlation values did not change significantly in the presence of Aβ, nor did the correlation difference between the experimental conditions. Values represent the mean ± SEM. *Wilcoxon matched-pairs sign rank test or paired Student’s *t*-test, *p* < 0.05.

We aimed to characterize the hippocampal network alterations in terms of cellular interactions using cross-correlation analysis (Kirkwood, 1979) among the elements recorded in the hippocampal CA1 (Figure 5). We obtained the cross-correlograms from pairwise analysis between elements recorded from each slice, where only correlations with peaks exceeding 5 SD of the noise correlation were computed, considered as functional connections, and included in correlation linkage matrices. In the presence of Aβ, the number of positive (193.3 ± 28.58 and 236.8 ± 58.23, for baseline and Aβ, respectively; *p* = 0.135; Figures 5A,B1, [+] left graph) and negative (5.40 ± 1.86 and 5.10 ± 2.77, for baseline and Aβ, respectively; *p* = 0.393. Figure 5B1, [₋] right graph) binary correlations did not change significantly. The strength of the interactions among the hippocampal elements was diverse under both experimental conditions, as revealed by the correlation linkage matrices (weighted correlations), where the weighted positive correlation values are indicated in a peak-representation map for both conditions (Figure 5A) and the sum of positive (+) and negative (₋) weighted correlation values (Figure 5B2). The values of the weighted positive (7.53 ± 1.47 and 9.49 ± 2.61, for baseline and Aβ, respectively; *p* = 0.120; Figures 5A,B2, [+] left graph) and weighted negative (−0.05 ± 0.02 and −0.05 ± 0.03, for baseline and Aβ, respectively; *p* = 0.461. Figure 5B2, [₋] right graph) correlations did not change significantly in the presence of Aβ. The average change in correlation indicated several positive and negative changes in the correlation values during Aβ treatment (*Δ* Correlation, Figure 5C). The absolute values displayed a significant increase during network reconfiguration in the presence of Aβ (0.0298 ± 0.0012 and 0.0289 ± 0.0017 for an increase [n = 1787] and a decrease [n = 1,164] in the absolute values, *p* = 0.002; Figure 5C, right graph). However, Cohen’s D statistics showed that the effect size between both groups is trivial or not significant (Cohen’s d = 0.017).

### 3.5 Aβ acutely modifies the hippocampal neuronal network configuration

The significantly depressed hippocampal network activity and simultaneous firing (activity clusters) of several hippocampal elements during the transition of activity induced by Aβ might involve changes in the topological configuration of the hippocampal network. For that reason, we evaluated different parameters to estimate features of the network architecture, including statistical metrics, centrality measures, and topological network classification using the small-world estimator and the Estrada index. These analyses were performed per slice, as the effective statistical n for network measurements is the number of slices that contain a specific number of units. The results of the statistical metrics showed that, in presence of Aβ, there were no significant differences in the number of connections between nodes (Average degree, *p* = 0.141; Table 1 and Supplementary Table 1), connections between groups of neurons (Density, *p* = 0.171; Table 1 and Supplementary Table 1), or network components (Connected component, *p* = 0.107; Table 1 and Supplementary Table 1). This also applied to the linear size of the network, given by the average distance between all pairs of connected nodes (Characteristic path length, *p* = 0.461; Table 1 and Supplementary Table 1), and to the average fraction of connections between nodes and their nearest neighbors (Clustering coefficient, *p* = 0.190; Table 1 and Supplementary Table 1). Furthermore, centrality measures were used to evaluate the importance of nodes within the network. However, in the presence of Aβ, none of these metrics displayed significant variations (Betweenness centrality, *p* = 0.268; Closeness centrality, *p* = 0.252; Eigenvector centrality, *p* = 0.415; and Eccentricity, *p* = 0.500; Table 1 and Supplementary Table 2).

**Table 1:** Estimation parameters of the network architecture features.

Parameter	Baseline	Aβ	*p* value
Statistical metrics			
Average degree	6.10 ± 0.68	7.07 ± 1.23	0.141
Density	0.10 ± 0.01	0.11 ± 0.01	0.171
Connected component	3.10 ± 0.46	4.00 ± 0.92	0.107
Characteristic path length	3.20 ± 0.19	3.21 ± 0.32	0.461
Clustering coefficient	0.48 ± 0.03	0.45 ± 0.04	0.190
Centrality measures			
Betweenness centrality	60.66 ± 5.92	55.73 ± 7.05	0.268
Closeness centrality	0.33 ± 0.02	0.34 ± 0.03	0.252
Eigenvector centrality	0.32 ± 0.02	0.33 ± 0.02	0.415
Eccentricity	5.39 ± 0.34	5.34 ± 0.65	0.500

Neither the statistical metrics nor the evaluation of the most important nodes in the network through the centrality measures showed any significant variations in their specific parameters. Data are expressed as mean ± SEM. The *p*-value is indicated for each parameter, obtained from a paired Student’s *t*-test or Wilcoxon matched-pairs sign rank test.

**Figure 6:**
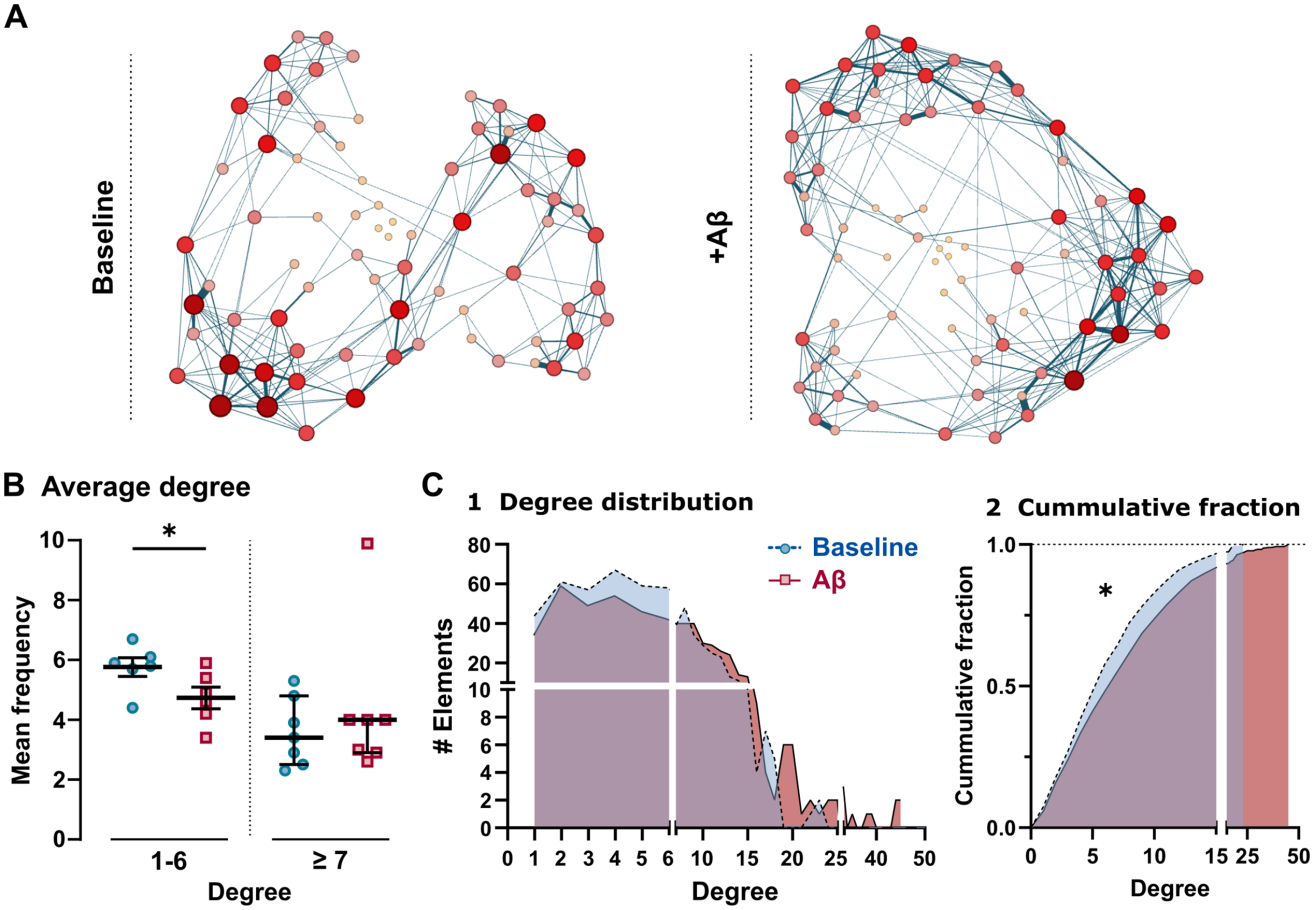
Degree distribution of the CA1 hippocampal network neuronal elements under Aβ exposure. **(A)** Graphical representation of the CA1 hippocampal network configuration under baseline and Aβ conditions, where each neuronal element is represented by a circle and functional links are represented by lines. The larger and redder the circle, the more connected the node. Thicker lines represent higher strength of connections between elements. **(B)** Degree values under baseline and Aβ conditions in the range of 1–6 and ≥7 degrees of the network elements. **(C)** The total (1) and cumulative (2) degree distribution of the network elements depicted by the specific frequency of each degree under baseline (blue area) and Aβ (red area) conditions. Notice the significant reduction in elements with a low degree value (in the range of 1–6) and the presence of hyperconnected nodes (>25 degree) under the Aβ condition. *Paired Student’s *t*-test or Kolmogorov–Smirnov test, *p* < 0.05.

Using statistical metrics, we also elucidated the configuration of the CA1 hippocampal network under baseline and Aβ conditions (Figure 6A; representation of one experiment in both conditions) using the built-in ForceAtlas layout algorithm (Gephi Consortium). Each node in this algorithm is represented by a circle, and the functional links are denoted by lines. The larger and redder the circle, the more connections it has, and thick lines represent highly correlated firing between the paired represented elements (connection strength). Note that the figure displays a different arrangement of the elements in both conditions, showing a tendency toward enhanced clustering of their activity, with the apparent very local increases in correlation strength under the Aβ condition (Figure 6A). Therefore, although we could not find any difference in the average value of the functional connections between the different neuronal elements (nodes) in the network (average degree), when we analyzed the probability distribution of these degrees over the whole network (degree distribution), we observed a significant reduction in the frequency of the nodes whose degree value ranges from 1–6 (5.77 ± 0.31 and 4.73 ± 0.36 for baseline and Aβ, respectively; *p* = 0.004; Figures 6B, 5C and Supplementary Table 3). Furthermore, even when the computation of the nodes whose degree value is ≥7 did not show any statistically significant changes (3.59 ± 0.43 and 4.34 ± 0.95 for baseline and Aβ, respectively; *p* = 0.296; Figure 6B), we observed a progressive increase in the number of hyperconnected nodes (around 20–40 connections) in the Aβ condition, compared with the baseline, which did not have many hyperconnected nodes (Figure 6C and Supplementary Table 3).

**Figure 7:**
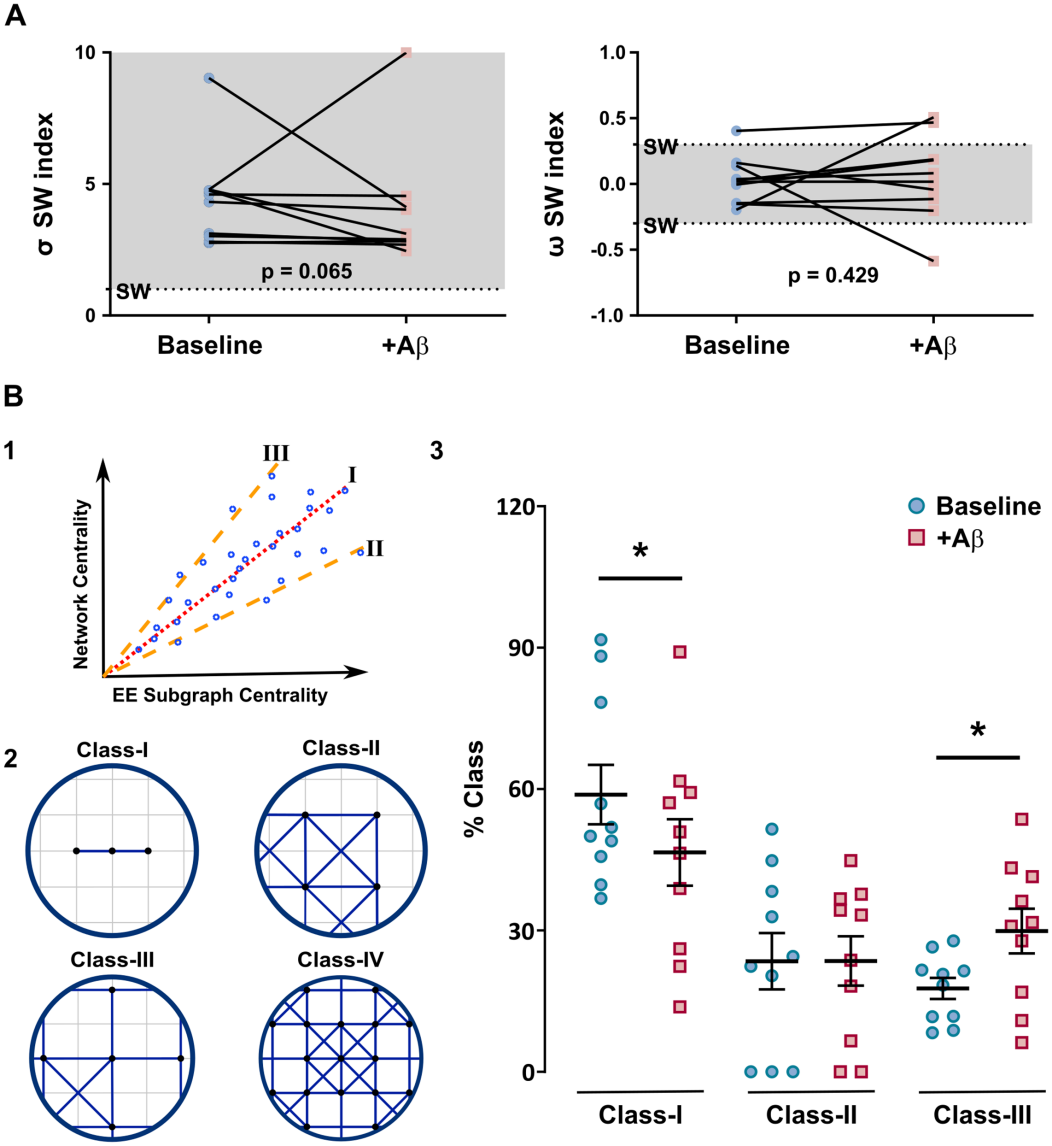
Topological analysis and classification of the network. **(A)** Individual values representing the sigma (left graph) and omega (right graph) values of the small-world metrics under baseline and Aβ conditions. **(B1)** The representation of the Estrada index classification criteria depicts the thresholds (dashed lines), which were set by calculating the variance of the distances between the ideal correlation (red dotted line) and the real correlation obtained from each node in the network. **(B2)** Representative illustrations of network configurations according to the Estrada index classification. Class I represents data that fall exactly on the ideal correlation line; class II corresponds to values of communicability below the lower bound; class III corresponds to correlations on the upper limit; and class IV is a combination of classes II and III. **(B3)** Graphical representation of the proportion (%) of each topological class according to the Estrada index classification of the baseline and Aβ network representations. Note that the sigma small-world index tends to decrease, suggesting small changes in the topological structure of the CA1 hippocampal network in the presence of acute Aβ. Furthermore, the Estrada index suggests that while baseline and Aβ networks maintained their class IV topology, there was a significant shift toward generating a less homogeneous network topology (class I) and increase in central nodes (class III). Values represent the mean ± SEM. *Paired Student’s *t*-test; *p* < 0.05.

After determining statistical metrics, we assessed the small-world properties by comparing the clustering coefficient and characteristic path length of 1,000 random and lattice networks with those of our experimental networks. The *σ* index (4.40 ± 0.58 and 3.95 ± 0.71 for baseline and Aβ conditions, respectively; *p* = 0.065; Figure 7A, left graph) and *ω* index (0.03 ± 0.06 and 0.05 ± 0.10 for baseline and Aβ conditions, respectively; *p* = 0.429; Figure 7A, right graph) denoted the typical values of the small-world property; that is, a highly clustered network with short path lengths (Watts and Strogatz, 1998). Besides, we observed a non-significant trend in which the sigma index of the Aβ network (Figure 7A) decreased with respect to the baseline condition, suggesting that the acute effects of Aβ treatment did not significantly affect the topological integrity measured by the small-world property.

In order to elucidate meaningful changes in the network arrangement associated with the Aβ treatment, we determined the network’s topological classification using the Estrada index (Estrada, 2011), as depicted in Figure 7B. We first used binary matrices following the original Estrada categorization of networks (Estrada, 2007). However, the results showed no distinctive pattern between baseline and Aβ conditions (data not shown). Consequently, we adjusted the topological classification method using the weighted correlation values. These values impact the spectral theory of this approach (Estrada, 2007), as previously utilized (Estrada, 2000), and allow a greater looseness in the limits of each class. We also set a new threshold by calculating the var*iance* of distances between the ideal correlation (red line) and the real correlation from each node in the network, thereby extending the classification margin for class I (dashed lines in Figure 7B1). Importantly, this distance corresponds to the eigenvector centrality value (y-axis) in the network. Thus, a conical region is defined by these new limits, obtained from the correlation values of the ideal line ± the determined threshold. This conical region represents the ideal correlation for each node (Figure 7B1). With this strategy, nodes can be classified to identify clearer tendencies among the topological classes and to characterize the different conditions of the neural networks. Hence, according to the new classification, nodes in this region are designated as class I (data are exactly on the ideal correlation line). Class II corresponds to values of communicability below the lower bound, class III to those above the upper limit, and class IV to a combination of classes II and III.

According to the topological classification, baseline and Aβ networks were identified as class IV (Ratio CII/CIII: 1.58 ± 0.58 [70% total] vs. 1.22 ± 0.54 [80% total]), respectively; *p* > 0.999 (Figure 7B2). In most cases, class IV structures are formed by highly connected neighborhoods. They also have certain regions that are weakly connected to the rest of the nodes. Therefore, class IV structures are preserved under the Aβ condition. On the other hand, we identified a significant topological difference by calculating the percentages of each class under both experimental conditions. In the baseline networks, we obtained the highest percentage for class I (58.83 ± 6.30%), followed by class II (23.48 ± 5.98%), and then class III (17.70 ± 2.25%), as depicted in Figure 7B3. These results indicate that the structure of the network under baseline conditions is more homogeneous (class I), with several connected neighborhoods (class II) and central nodes (Figure 7B2, inset representations). For networks under the Aβ condition, we obtained 46.57 ± 7.04% for class I, 23.53 ± 5.23% for class II, and 29.89 ± 4.73% for class III (Figure 7B3). Note that class II percentages in both conditions exhibited a slight change (*p* = 0.494), whereas class I and III percentages exhibited a statistically significant change (20.8% decrease, *p* = 0.026; 40.8% increase, *p* = 0.044, respectively, under the Aβ condition; Figure 7B3). This finding suggests that the networks became less homogeneous under the Aβ condition (reduction in class I compared with the baseline condition). Therefore, the number of central nodes (class III) increased (Figures 7B2,B3). This tendency towards enhanced clustering, as described by the Estrada index, correlates with the distinct arrangement of elements in both conditions (Figure 6A) and with their degree distribution (Figures 6B,C).

## 4 Discussion

Neurodegenerative diseases, including AD, have been associated with alterations in the activity of functionally connected neuronal networks (Kowalski et al., 2001; Anticevic et al., 2012; Bakker et al., 2012; Sperling et al., 2009; Allen et al., 2007). Pathologically high concentrations of Aβ are a benchmark for these changes (Peña-Ortega, 2013). In this work, we used MEA recordings to evaluate changes in functional and structural network properties in the presence of Aβ. We assessed alterations in the activity and connection strength of the elements that integrate the network by analyzing the firing correlation between each pair of connected cells. We found that Aβ reduced CA1 hippocampal circuit functionality, expressed as a decrease in firing frequency of network cells. This finding is correlated with enhanced bursting activity and a change in the network’s topological reconfiguration, according to our adaptation of the Estrada index (Estrada, 2007). However, we found no significant changes in the correlation values that represent the average number of functional links among elements, the average change in the strength of this connectivity, and the difference in total correlation changes between both conditions.

The effects of Aβ on the stability of neuronal networks involve several physiological changes at the cellular level. These changes progressively impact neuronal network configuration and function (Alcantara-Gonzalez et al., 2019; Colom et al., 2010; Alcantara-Gonzalez et al., 2021; Palop and Mucke, 2010; Palop et al., 2007; Villette et al., 2012). In this study, we reported a reduction in the activity of hippocampal elements induced by Aβ, which is similar to what has been previously reported using the same approach (MEAs) in hippocampal cultured neurons (Varghese et al., 2010; Alhebshi et al., 2013), other cortical neuronal networks (Charkhkar et al., 2015; Gortz et al., 2009), and with patch clamp or other recordings in hippocampal cells (Villette et al., 2010; Varghese et al., 2010; Yun et al., 2006; Haghani et al., 2012). Regarding the hippocampal glutamatergic cells, some studies show that Aβ is able to modify its activity towards an increment (Minkeviciene et al., 2009; Ren et al., 2014; Juhasz et al., 2009; Busche et al., 2012; Alcantara-Gonzalez et al., 2025) or a reduction (Alhebshi et al., 2013; Haghani et al., 2012). Besides, Aβ also causes the cellular desynchronization of action potentials and reduces their capacity of generating subthreshold oscillations in the theta range, which underlies the inhibition of network activity (Pena et al., 2010; Alcantara-Gonzalez et al., 2019; Villette et al., 2012; Kurudenkandy et al., 2014). With respect to interneurons, it has been described that they are particularly sensitive to Aβ-induced alterations, like the impairment in their firing pattern (Villette et al., 2010; Palop et al., 2007; Rubio et al., 2012; Hazra et al., 2013; Hijazi et al., 2020a; Hijazi et al., 2020b), which is reflected in the disruption of neural network dynamics (Verret et al., 2012; Villette and Dutar, 2017; Albuquerque et al., 2015; Baglietto-Vargas et al., 2010).

Consistent with this, when we analyzed the individual changes in firing frequency, we identified two subpopulations of cells. One group that reduced their firing frequency, and another that increased it. This same dichotomy of abnormally hyperactive and hypoactive neurons has also been found in the cortex of hAPP/PS1 transgenic mice (Busche et al., 2008), where the cells that were closer to the Aβ plaques presented the highest activity, presumably due to an impaired synaptic inhibition caused by Aβ. Likewise, other mechanisms leading to Aβ-induced neuronal hyperactivity involve alterations in neuronal electrophysiological properties (Hou et al., 2009), inhibition of K^+^ currents (Qi et al., 2004; Chen, 2005), increases in the persistent sodium current (I_NaP_) (Ren et al., 2014), disruption of intracellular Ca^2+^ regulation (Ueda et al., 1997; Rovira et al., 2002), and the induction of cation-selective channels in the cellular membrane (Arispe et al., 1993; Kawahara and Kuroda, 2000). Nevertheless, our main finding was the reduction of average activity, which reflects the diminished firing of almost 62% of the recorded cells. This effect may involve some Aβ-induced mechanisms, such as the inhibition of synaptic currents (Kamenetz et al., 2003; Chang et al., 2006; Nimmrich et al., 2008), differentially inhibiting and potentiating the NMDA and GABA_A_ receptor-mediated cell currents, respectively (Zhang et al., 2009), decreasing the glutamatergic receptors expression (Hsieh et al., 2006; Almeida et al., 2005; Roselli et al., 2005; Snyder et al., 2005), reducing dendritic spines and electrophysiological active synapses (Shankar et al., 2007; Tsai et al., 2004), and the remodeling of inhibitory synaptic inputs causing the enhancement of inhibition (Palop et al., 2007). These alterations in cellular activity may impact network function, causing dysrhythmias, including aberrant network synchronization and simultaneous firing of several hippocampal elements, as reported in some models of AD (Palop et al., 2007). Our data are consistent with these findings, as we observed enhanced bursting activity. More experiments should be done to elucidate the identity of the cells that change their activity under our experimental conditions.

The bursting activity induced by Aβ and the change in neuronal activity (hyperactive and hypoactive) may involve the functional or anatomical remodeling of both excitatory and inhibitory synapses. Additionally, the increased excitatory network activity induced by Aβ may lead to synaptic depression through homeostatic plasticity compensatory mechanisms that may differentially affect several types of neurons (Palop and Mucke, 2010). Interestingly, despite decreased spontaneous synaptic activity, the average firing correlation among elements did not show any differences, as reported elsewhere (de la Rocha et al., 2007; Cohen and Kohn, 2011). Instead, we observed that the network’s correlation was preserved, suggesting that some adjustments occur in the neural circuit to maintain its physiological activity. Brain circuits can modulate their network configuration to sustain neuronal circuit function. They achieve this by regulating a set of homeostatic plasticity mechanisms, such as the expression of surface receptors and the tuning of synaptic strengths, which may be regulated to promote the stability of the constant firing rate for each type of neuron (Turrigiano, 2012; Turrigiano et al., 1998; Turrigiano, 1999). Our findings suggest that these mechanisms, which compensate for the global reduction in firing frequency, are characterized by an increase in synchronic cell activation in certain time bins, preserving the correlation strength in the circuit. To our consideration, the increased occurrence of synchronized burst, increased hyperconnected nodes and the increase in firing of some elements are secondary changes to the initial Aβ-reduction in firing frequency of some neuronal elements in the network and the reduction in LFP power. These mechanisms can constitute one of many others that compensate for changes in the network functional correlations (Turrigiano, 2012; Caporale and Dan, 2008; Desai et al., 1999; Vogels et al., 2011). Regarding the increased number of neuronal elements with higher degree distribution we observed in presence of Aβ can potentially constitute hub cells. It is known that, in pathological conditions, the generation of highly interconnected neurons greatly increases network activity, resulting in a hyperexcitable circuit (Morgan and Soltesz, 2008; Stam, 2024). Accordingly, hub neurons also serve as correlation stabilizers as hyperconnected nodes exert an increased influence on population-wide coordination (Bonifazi et al., 2009), and when hub neurons are disturbed, the correlations are affected (Bonifazi et al., 2009; Uzel et al., 2022; Shimono and Beggs, 2015; Khambhati et al., 2019). Under our experimental conditions, these hub cells can either be produced by the lack of inhibitory inputs, as ~75% of PINs reduced their activity; or they also can be constituted by those PINs that increased their firing (Bonifazi et al., 2009). Therefore, the lack of changes in functional correlations can be explained by the increased firing frequency of a subset of hyperactive excitatory elements, as reported by Busche, Eichhoff (Busche et al., 2008) near the source of Aβ; or by a direct influence of the presence of hub cells (Bonifazi et al., 2009). This increase in hub neurons may be necessary to maintain a shared drive as neurons still receive proportionally similar synaptic drive, which is relevant to pairwise correlations (Uzel et al., 2022). In this way, correlations can be preserved even when average firing rates decrease, which can be a homeostatic plasticity mechanism where synaptic scaling preserves relative synaptic weights (Turrigiano, 2012; Turrigiano et al., 1998; Turrigiano and Nelson, 2004; Abbott and Nelson, 2000). All these speculative explanations need proper experimental validation.

However, while neuron synchronization within a network is important for effective information processing, it can also reflect aberrant network dynamics under pathological or non-physiological conditions. These dynamics could be implicated in learning and memory impairment. They may also contribute to an increased risk for seizure-like activity and establish a pro-epileptic brain state, as has been reported in AD patients and animal models with an increased incidence of epileptic seizures (Colom et al., 2010; Palop et al., 2007; Minkeviciene et al., 2009; Friedman et al., 2012; Rao et al., 2009; Palop and Mucke, 2009; Ziyatdinova et al., 2011; Colom, 2006). In fact, we have previously reported that after three weeks of intracerebroventricular injection of Aβ, there are some synaptic changes that correlate with alterations in the oscillatory pattern of the hippocampal area and facilitate the generation of acutely induced seizures (Alcantara-Gonzalez et al., 2019).

In order to characterize functional organization in complex networks, we evaluated connectivity properties using classical approaches in the framework of graph formalism, applying statistical metrics and centrality measures that assess node interactions and information transfer efficiency between two network regions (Feldt et al., 2011; Vecchio et al., 2016; Bullmore and Sporns, 2009; Boccaletti et al., 2006). We also determined the small-world property to evaluate the stability of our network. However, we observed no changes in these parameters, suggesting that network’s integrity remains robust with only discrete alterations in the topological structure of the CA1 hippocampal network associated with the short-term effects of Aβ on neuronal activity. This is logical, as only acute exposure to Aβ was evaluated. Our findings are different from reports in which a change in neuronal activity—in this case, an enhancement—is accompanied by a loss in the small-world network topology (Srinivas et al., 2007). We observed no changes in the small-world property or a reduction in overall activity possibly because of the subsequent appearance of simultaneous firing in several cells, which might represent an enhancement in the connectivity between the recorded units and probably compensating the small-world structure. Therefore, we used the Estrada index as a complementary method to identify changes in terms of topological descriptors. Thus, when we elucidated the neuronal network’s topological distribution of classes, we observed that, in the presence of Aβ, the network leans towards a less homogeneous structure due to the reduction in the ordered class I distribution and the emergence of an increasingly hyperconnected core with a neighborhood that has low-density connections (class III). This general topological network distribution, depicted in Figure 4A, tends to exhibit a highly localized clustering activity with increased correlation strength under the Aβ condition, suggesting a reduction in the communication between distant clusters. This finding is consistent with the notion that AD is a syndrome of disconnection among the neuronal networks of the brain (Delbeuck et al., 2003; He et al., 2009). Research on the small world properties of networks (Stam and Reijneveld, 2007; Bassett and Bullmore, 2006) has found evidence of this disconnection when comparing between different brain structures (Sanz-Arigita et al., 2010; Stam et al., 2009). Therefore, a lack of connectivity may contribute to the learning and memory impairments observed during mild cognitive impairment in AD. During this stage of AD, the concentration of Aβ rises, resulting in hyperexcitability (Vossel et al., 2017). To the best of our knowledge, this is the first time that the Estrada index has been used to describe a more complex neuronal network. Therefore, further research should be conducted with this index to validate our data.

In conclusion, using a multielectrode recording *in vitro* approach, we demonstrated that acute exposure to a low concentration of Aβ primarily induced a general decrease in the spontaneous activity of the CA1 hippocampal neuronal circuit, reflecting reduced activity in most cells. However, another subset of cells exhibited synchronous firing activity. We suggest that the enhanced activity of these cells may indicate an adaptive homeostatic plasticity mechanism that could be responsible for maintaining a baseline level of activity in the circuit, allowing correlations among elements to remain constant. We believe that sustaining this type of activity in the long term could possibly lead to enhanced circuit hyperexcitability and, eventually, memory impairment (Zhang et al., 2025; Corriveau-Lecavalier et al., 2024; Styr and Slutsky, 2018). Besides, there are some discrete but important alterations in network topology associated with the acute effects of Aβ, which may correlate with the modifications in circuit activity. As far as we know, this is the first topological description of a large biological neuronal network using the Estrada index approach, opening avenues for research in the field of network topology.

As a final consideration, despite the key findings regarding the alterations in the CA1 hippocampal network we presented in this work, this study has some limitations and lays the foundation for further characterization of these changes. In this work, we used an oligomerized solution of synthetic Aβ which certainly has proven validity in a wide range of studies (Salgado-Puga and Pena-Ortega, 2015; Salgado-Puga et al., 2015; Peña-Ortega, 2013; Varshavskaya et al., 2022; Weidner et al., 2011). However, it would be relevant to know whether the alteration in network stability described in our study can be reproduced when using a more naturally derived oligomeric Aβ source, like conditioned media-derived Aβ (Hahn et al., 2011; Lewis et al., 2001; Pavoni et al., 2018; Reed et al., 2011). It would also be very relevant to evaluate the time-course and the concentration-dependent alterations induced by Aβ to elucidate more discrete changes in the hippocampal network structure and track the development of these processes in time and in response to Aβ levels (Koppensteiner et al., 2016; Calabrese et al., 2007; Parodi et al., 2010; Estus et al., 1997). Furthermore, even though 5 SD noise correlations value is broadly used in the field (Nieto-Posadas et al., 2014; Ostojic et al., 2009) to avoid any confounding random correlation, the development of more detailed studies and more sensitive analysis to explore synaptic connectivity between neurons with <5 SD noise correlations should be performed to detect weaker but meaningful connections, as they might be underestimated in this work. Exploring the Aβ effects in both sexes is relevant for a broader data interpretation. In this study, we simplified our experimental approach by using only males. However, we acknowledge that data obtained from males cannot be reliably extrapolated to females, as biological differences due to variations in genetics, hormones, metabolism, etc. may lead to different outcomes. Consequently, including females improves the robustness, validity and reproducibility of scientific studies, which better reflects the biological reality (Beery and Zucker, 2011). We propose that these changes need to be further evaluated in females and larger samples as well. Finally, we agreed that there is a technical limitation regarding the network metrics and estimation of small-world indexes, as they may be underpowered due to low node count (Telesford et al., 2011; Martin and Niemeyer, 2021; Tuhin et al., 2023). However, there are some parameters that are stable within sizes between 50 and 100 nodes. Although this represents a technical limitation, the identification of nodes and their mutual relationship was carried out systematically using the routines implemented to accurately recover the behavior of these networks. We acknowledge there is some degree of uncertainty about our conclusions under these experimental conditions, and we propose the use of larger networks should be considered for future experiments.

## Data Availability

The original contributions presented in the study are included in the article/Supplementary material, further inquiries can be directed to the corresponding authors.
