# Supplementary material for: Amyloid-β acute exposition affects the CA1 hippocampal network activity and its topological organization, evaluated with multielectrode arrays
Source: Front Dement. 2026 Mar 6;5:1738954. doi: 10.3389/frdem.2026.1738954 (PMC13002378; doi:10.3389/frdem.2026.1738954)
Supplement: Supplementary file 1 [file Table_1.docx]

**Supplementary tables.**

**Supplementary table 1.** **Estimation parameters of network architecture for each individual experiment.** Statistical metrics data obtained from all the network elements and reported for each independent slice are presented for every variable evaluated.

| ***Statistical metrics*** | |  |  |  |  |  |  |  |
| --- | --- | --- | --- | --- | --- | --- | --- | --- |
| **Exp/Condition** | **Nodes (N)** | | **Links (K)** | **Average degree** | **Density** | **Connected component** | **Characteristic path length** | **Clustering coefficient** |
| **C1 Baseline** | 58 | | 76 | 2.621 | 0.046 | 5 | 4.415 | 0.249 |
| **C2 Baseline** | 60 | | 156 | 5.200 | 0.088 | 3 | 3.170 | 0.474 |
| **C3 Baseline** | 49 | | 177 | 7.224 | 0.151 | 1 | 2.803 | 0.553 |
| **C4 Baseline** | 68 | | 337 | 9.912 | 0.148 | 1 | 2.694 | 0.62 |
| **C5 Baseline** | 73 | | 217 | 5.945 | 0.083 | 4 | 3.294 | 0.487 |
| **C6 Baseline** | 65 | | 211 | 6.492 | 0.101 | 5 | 3.853 | 0.507 |
| **C7 Baseline** | 88 | | 326 | 7.409 | 0.085 | 2 | 2.997 | 0.524 |
| **C8 Baseline** | 36 | | 65 | 3.611 | 0.103 | 3 | 3.050 | 0.386 |
| **C9 Baseline** | 56 | | 218 | 7.786 | 0.142 | 3 | 2.374 | 0.512 |
| **C10 Baseline** | 63 | | 150 | 4.762 | 0.077 | 4 | 3.344 | 0.436 |
|  |  | |  |  |  |  |  |  |
| **C1 Aβ** | 58 | | 76 | 2.621 | 0.046 | 4 | 5.081 | 0.155 |
| **C2 Aβ** | 60 | | 147 | 4.900 | 0.083 | 2 | 3.124 | 0.375 |
| **C3 Aβ** | 49 | | 179 | 7.306 | 0.152 | 1 | 2.696 | 0.556 |
| **C4 Aβ** | 68 | | 345 | 10.147 | 0.151 | 2 | 2.683 | 0.578 |
| **C5 Aβ** | 73 | | 287 | 7.863 | 0.109 | 5 | 2.663 | 0.492 |
| **C6 Aβ** | 65 | | 220 | 6.769 | 0.106 | 11 | 2.355 | 0.329 |
| **C7 Aβ** | 88 | | 696 | 15.818 | 0.182 | 2 | 2.075 | 0.502 |
| **C8 Aβ** | 36 | | 50 | 2.778 | 0.079 | 6 | 4.665 | 0.563 |
| **C9 Aβ** | 56 | | 216 | 7.714 | 0.140 | 3 | 2.764 | 0.561 |
| **C10 Aβ** | 63 | | 152 | 4.825 | 0.078 | 4 | 3.945 | 0.353 |

**Supplementary table 2. Estimation parameters of network architecture for each individual experiment.** Centrality measures data obtained from all the network elements and reported for each independent slice are presented for every variable evaluated.

| ***Centrality measures*** | |  |  |  |
| --- | --- | --- | --- | --- |
| **Exp/Condition** | **Betweenness centrality** | | **Closeness centrality** | **Eccentricity** |
| **C1 Baseline** | 75.414 | | 0.258 | 7.397 |
| **C2 Baseline** | 59.783 | | 0.310 | 5.133 |
| **C3 Baseline** | 43.265 | | 0.365 | 4.694 |
| **C4 Baseline** | 56.750 | | 0.379 | 4.309 |
| **C5 Baseline** | 75.904 | | 0.299 | 5.671 |
| **C6 Baseline** | 77.723 | | 0.281 | 6.339 |
| **C7 Baseline** | 84.898 | | 0.342 | 6.193 |
| **C8 Baseline** | 31.944 | | 0.321 | 5.028 |
| **C9 Baseline** | 35.107 | | 0.414 | 3.732 |
| **C10 Baseline** | 65.841 | | 0.292 | 5.365 |
|  |  | |  |  |
| **C1 Aβ** | 104.483 | | 0.195 | 9.466 |
| **C2 Aβ** | 60.567 | | 0.321 | 4.833 |
| **C3 Aβ** | 40.694 | | 0.380 | 4.510 |
| **C4 Aβ** | 54.721 | | 0.373 | 4.574 |
| **C5 Aβ** | 53.438 | | 0.361 | 4.151 |
| **C6 Aβ** | 30.954 | | 0.366 | 3.385 |
| **C7 Aβ** | 45.705 | | 0.487 | 3.068 |
| **C8 Aβ** | 38.889 | | 0.312 | 7.306 |
| **C9 Aβ** | 45.071 | | 0.359 | 4.643 |
| **C10 Aβ** | 82.730 | | 0.251 | 7.508 |

**Supplementary table 3. Degree distribution.** Degree distribution data from each independent slice.

| ***Degree distribution (Elements with a specific degree; frequency of nodes).*** | | | | | | | |
| --- | --- | --- | --- | --- | --- | --- | --- |
| **Condition / Degree** | **1** | **2** | **3** | **4** | **5** | **6** | **7** |
| **C1 Baseline** | 14 | 9 | 17 | 10 | 2 | 2 | 1 |
| **C2 Baseline** | 5 | 6 | 3 | 8 | 11 | 8 | 5 |
| **C3 Baseline** | 2 | 1 | 1 | 4 | 1 | 6 | 13 |
| **C4 Baseline** | 2 | 1 | 1 | 2 | 7 | 7 | 2 |
| **C5 Baseline** | 2 | 10 | 5 | 6 | 7 | 11 | 6 |
| **C6 Baseline** | 6 | 9 | 4 | 7 | 3 | 5 | 2 |
| **C7 Baseline** | 5 | 5 | 5 | 12 | 9 | 8 | 4 |
| **C8 Baseline** | 1 | 10 | 7 | 6 | 5 | 1 | 2 |
| **C9 Baseline** | 3 | 2 | 3 | 3 | 8 | 5 | 0 |
| **C10 Baseline** | 4 | 8 | 11 | 9 | 6 | 5 | 4 |
| **Condition / Degree** | **8** | **9** | **10** | **11** | **12** | **≥13** |  |
| **C1 Baseline** | 0 | 0 | 0 | 0 | 0 | 0 |  |
| **C2 Baseline** | 1 | 6 | 3 | 1 | 0 | 1 |  |
| **C3 Baseline** | 8 | 4 | 4 | 3 | 1 | 1 |  |
| **C4 Baseline** | 8 | 4 | 6 | 3 | 6 | 19 |  |
| **C5 Baseline** | 5 | 6 | 4 | 3 | 3 | 2 |  |
| **C6 Baseline** | 4 | 3 | 4 | 3 | 4 | 8 |  |
| **C7 Baseline** | 10 | 4 | 3 | 3 | 9 | 10 |  |
| **C8 Baseline** | 1 | 0 | 0 | 1 | 0 | 0 |  |
| **C9 Baseline** | 6 | 4 | 4 | 5 | 0 | 11 |  |
| **C10 Baseline** | 5 | 3 | 1 | 3 | 0 | 1 |  |

|  | | | | | | | |
| --- | --- | --- | --- | --- | --- | --- | --- |
| **Condition / Degree** | **1** | **2** | **3** | **4** | **5** | **6** | **7** |
| **C1 Aβ** | 8 | 22 | 11 | 8 | 3 | 1 | 2 |
| **C2 Aβ** | 5 | 6 | 3 | 12 | 9 | 12 | 4 |
| **C3 Aβ** | 2 | 0 | 5 | 3 | 3 | 5 | 7 |
| **C4 Aβ** | 1 | 1 | 1 | 2 | 3 | 3 | 5 |
| **C5 Aβ** | 4 | 5 | 2 | 5 | 6 | 3 | 1 |
| **C6 Aβ** | 1 | 4 | 4 | 2 | 4 | 3 | 8 |
| **C7 Aβ** | 0 | 2 | 1 | 2 | 5 | 6 | 3 |
| **C8 Aβ** | 4 | 9 | 9 | 6 | 3 | 2 | 0 |
| **C9 Aβ** | 3 | 1 | 7 | 3 | 2 | 2 | 8 |
| **C10 Aβ** | 6 | 9 | 6 | 11 | 8 | 5 | 2 |
| **Condition / Degree** | **8** | **9** | **10** | **11** | **12** | **≥13** |  |
| **C1 Aβ** | 0 | 0 | 0 | 0 | 0 | 0 |  |
| **C2 Aβ** | 3 | 2 | 0 | 3 | 0 | 0 |  |
| **C3 Aβ** | 5 | 7 | 5 | 5 | 0 | 2 |  |
| **C4 Aβ** | 11 | 5 | 4 | 5 | 6 | 20 |  |
| **C5 Aβ** | 6 | 10 | 6 | 3 | 5 | 13 |  |
| **C6 Aβ** | 3 | 7 | 5 | 4 | 3 | 7 |  |
| **C7 Aβ** | 5 | 5 | 4 | 2 | 6 | 46 |  |
| **C8 Aβ** | 0 | 0 | 0 | 0 | 0 | 0 |  |
| **C9 Aβ** | 3 | 3 | 3 | 5 | 5 | 9 |  |
| **C10 Aβ** | 4 | 1 | 3 | 2 | 1 | 2 |  |
